# Malignant ascites in pancreatic cancer: Pathophysiology, diagnosis, molecular characterization, and therapeutic strategies

**DOI:** 10.3389/fonc.2023.1138759

**Published:** 2023-03-16

**Authors:** Margaret Y. Han, Erkut H. Borazanci

**Affiliations:** ^1^Department of Biosciences, Rice University, Houston, TX, United States; ^2^Department of Oncology, HonorHealth Research Institute, Scottsdale, AZ, United States

**Keywords:** pancreatic cancer, malignant ascites, peritoneal carcinomatosis (PC), metastasis, chemotherapy, paracentesis

## Abstract

Malignant ascites is the accumulation of fluid in the peritoneum as a result of advanced cancer and often signifies the terminal phase of the disease. Management of malignant ascites remains a clinical challenge as symptom palliation is the current standard of cure. Previously, studies examining malignant ascites largely focused on ovarian and gastric cancer. In recent years, there has been a significant increase in research on malignant ascites in pancreatic cancer. Malignant ascites is usually diagnosed based on positive cytology, but cytology is not always diagnostic, indicating the need for novel diagnostic tools and biomarkers. This review aims to summarize the current understanding of malignant ascites in pancreatic cancer and the recent advances in the molecular characterization of malignant ascites fluid from patients with pancreatic cancer including analysis of soluble molecules and extracellular vesicles. Current standard of care treatment options such as paracenteses and diuretics are outlined along with new emerging treatment strategies such as immunotherapy and small-molecule based therapies. New potential investigative directions resulting from these studies are also highlighted.

## Introduction

Pancreatic cancer is a highly lethal disease with a 5-year survival rate of <11% (1). It is currently the third leading cause of cancer death in the United States and is projected to be the second by 2026 after lung cancer (2, 3). Pancreatic ductal adenocarcinoma (PDAC) which accounts for 90% of all pancreatic cancer cases is among the most lethal of all cancers (4). Surgical resection offers the only hope for cure for patients with PDAC but, unfortunately, is only applicable in 10-15% of patients. Prognosis remains very poor with a 5-year survival of < 1% for patients with advanced metastatic disease (5). One main factor that contributes to the poor prognosis for patients with late stage PDAC is its resistance to chemotherapy and targeted therapies including immunotherapies. The current standard of care therapies such as FOLFIRNOX and gemcitabine plus nab-paclitaxel only extend patient survival by 2-6 months (6–8). One important factor that affects the quality of life and significantly reduces the survival of patients with PDAC is the development of malignant ascites (MA).

Malignant ascites (MA) is defined as the accumulation of fluid in the peritoneal cavity due to cancer that causes troublesome symptoms such as pain, loss of appetite, dyspnea, nausea, and reduced mobility (9). It accounts for approximately 10% of all ascites cases and is prevalent in ovarian, colorectal, pancreatic, gastric, and primary peritoneal cancers (10). Since MA is predominantly associated with ovarian cancer and breast cancer, there is a higher occurrence of MA in women compared to men (11). Around 20% of MA cases have an unknown primary tumor, and 50% of cases present with ascites at initial diagnosis (12). A retrospective study of 209 patients of 17 different cancer types finds that MA is associated with advanced stage or metastatic disease and is a poor prognostic sign with a reported patient survival of less than 6 months upon presentation (11).

While the onset of MA is associated with reduced quality of life and a poor prognosis, there remains no generally accepted evidence-based guidelines for treatment (13). Additionally, there are no preventive measures for MA development due to a lack of clinical predictors. With a wide range of symptoms including abdominal distension, impaired mobility and respiration, and swelling of limbs, MA requires prompt management focusing not only on symptomatic relief but reduction of disease and recurrence. As Saif et al. suggest, individualized treatment is the logical approach to treating patients with MA (13). However, the majority of MA treatments aim towards the palliation of symptoms as there are few effective therapeutic treatments. Thus, new biomarkers and therapeutic targets for more effectively treating patients with MA are urgently needed. This review describes the current state of the characterization and treatment of malignant ascites in PDAC patients while highlighting the recent advances in molecular profiling and novel therapeutics development to guide more effective and individualized treatment of MA beyond simple palliation of symptoms.

## Pathophysiology and clinical manifestation of malignant ascites in PDAC

The pathophysiology of malignant ascites in PDAC and other cancers is complex and multifactorial and is yet to be completely understood. Our current understanding of the mechanism of MA formation mainly comes from studies in ovarian and other non-PDAC tumor types. The main physiological factor of MA development is the increased permeability of tumor vessels causing forced production and release of peritoneal fluid (14). Ascites fluid from patients with peritoneal carcinomatosis (PC) has a positive cytology with elevated protein concentration and low (<1.1 g/dL) serum-ascites albumin gradient (SAAG) (15). The elevated protein concentration indicates an alteration in vascular permeability allowing large molecules (i.e., proteins) to accumulate in the intraperitoneal space. This increased permeability has been shown to be caused by marked neovascularization of the parietal peritoneum and glycoprotein production (16). In a study by Garrison and colleagues using a rat breast cancer MA model, infusion of cell-free malignant ascites into the intraperitoneal space resulted in an increase in edema formation in omental vessels, indicating there exists a tumor-induced factor(s) in the fluid that alters vessel permeability and promotes the formation of MA (17). Furthermore, the levels of vascular endothelial growth factor (VEGF) which allows movement of molecules across the vascular endothelium in both normal physiological and pathological disease states were found to be elevated in MA compared to benign ascites and is believed to play an important role in altering vascular permeability and tumor growth (18). One other factor that contributes to the formation of MA is lymphatic obstruction (16). In healthy individuals, lymphatic drainage and differences in oncotic pressure allow for fluid reabsorption. In a mouse MA model for ovarian cancer, obstruction of lymphatic drainage was found to prevent peritoneal fluid absorption and lead to the formation of MA (19). In pancreatic cancer, portal hypertension (PH) induced by portal vein obstruction due to direct tumor invasion and extraluminal compression can also lead to the formation of ascites (20). Although ascites caused by PH can be cytology negative, in many cases they have a positive cytology with evidence of peritoneal carcinomatosis (21). Approximately 23% of ascites cases are associated with PH in pancreatic cancer, which is one of the highest cancer types studied (21). Overall, up to 30% of patients with PDAC develop MA (22).

The clinical manifestation of MA in patients with PDAC is similar to that of patients with other cancer types such as ovarian and gastric cancer. Despite this similarity, overall survival (OS) differs with MA of ovarian origin having better median survival than that of gastrointestinal (GI) origin including pancreatic cancer (11). One reason for this difference is because patients with GI cancers are more likely to have liver metastases which have been associated with significantly poor survival. Frequently reported symptoms in PDAC patients include abdominal distention/discomfort, shortness of breath, weight gain, nausea, and vomiting (23). In a study with 180 PDAC patients who presented/developed ascites, Hicks et al. reported a median overall survival of 1.8 months after ascites development (23). These results are supported by several studies with smaller cohort sizes. For instance, both Zervos et al. (24) and DeWitt et al. (25) reported a median OS of ~ 2 months after ascites development whereas Takahara et al. (22) reported an OS of 47 days regardless of the time of onset. In a case-control study, Baretti et al. confirmed that PDAC patients with ascites had a higher risk of death compared to patients without ascites (OS = 10.2 vs. 15.2 months, P < 0.001) (26). Alshuwaykh et al. found that patients with pancreatic cancer with evidence of PC had higher 1 and 5-year mortality rates compared to those without PC (68% vs. 33%, p = 0.04 and 57% vs. 17%, p = 0.02, respectively) (21).

## Diagnosis of MA

In addition to malignancy, a number of other conditions can also cause the formation of ascites including cirrhosis, kidney failure, congestive heart failure, nephrosis, and pancreatitis. To differentiate benign ascites from malignant ascites, cellular and/or molecular analyses of the fluid are necessary as physical exams or radiographic techniques alone are not able to distinguish between the two. As noted above, malignant ascites fluid usually has a positive cytology with elevated protein concentrations and low SAAG (27). However, cytology is not diagnostic in some cases and SAAG can be insensitive and non-specific, which requires additional analyses to accurately diagnose MA in patients with PDAC. In a retrospective analysis of 62 patients with PDAC who presented or developed ascites during the course of their disease and had their ascites fluid analyzed only 36 (58%) had positive cytology and the majority (82%) of the patients had a SAAG ≥ 1 (23). This highlights one of the challenges in managing PDAC patients with ascites as multiple paracentesis may be necessary to confirm the presence of malignancy. To this end, Han et al. reported that mutations identified in tumor DNA isolated from ascites fluid from patients with ovarian cancer agree with those identified in the corresponding primary tumor tissues, which provides a potentially new tool for diagnosing malignant ascites (28). In addition, Li et al. report that combining cytological tests with telomerase activity assay significantly enhances the differential diagnosis between malignant and non-malignant ascites (29). Cytokines such as Interleukin 6 in ascites have also been found to have higher sensitivity and specificity as a diagnostic marker for ovarian cancer (30). These potential biomarkers have yet to be studied in PDAC and their utility in diagnosing MA in patients with PDAC needs future exploration.

## Molecular characterization of malignant ascites in PDAC

**Figure 1:**
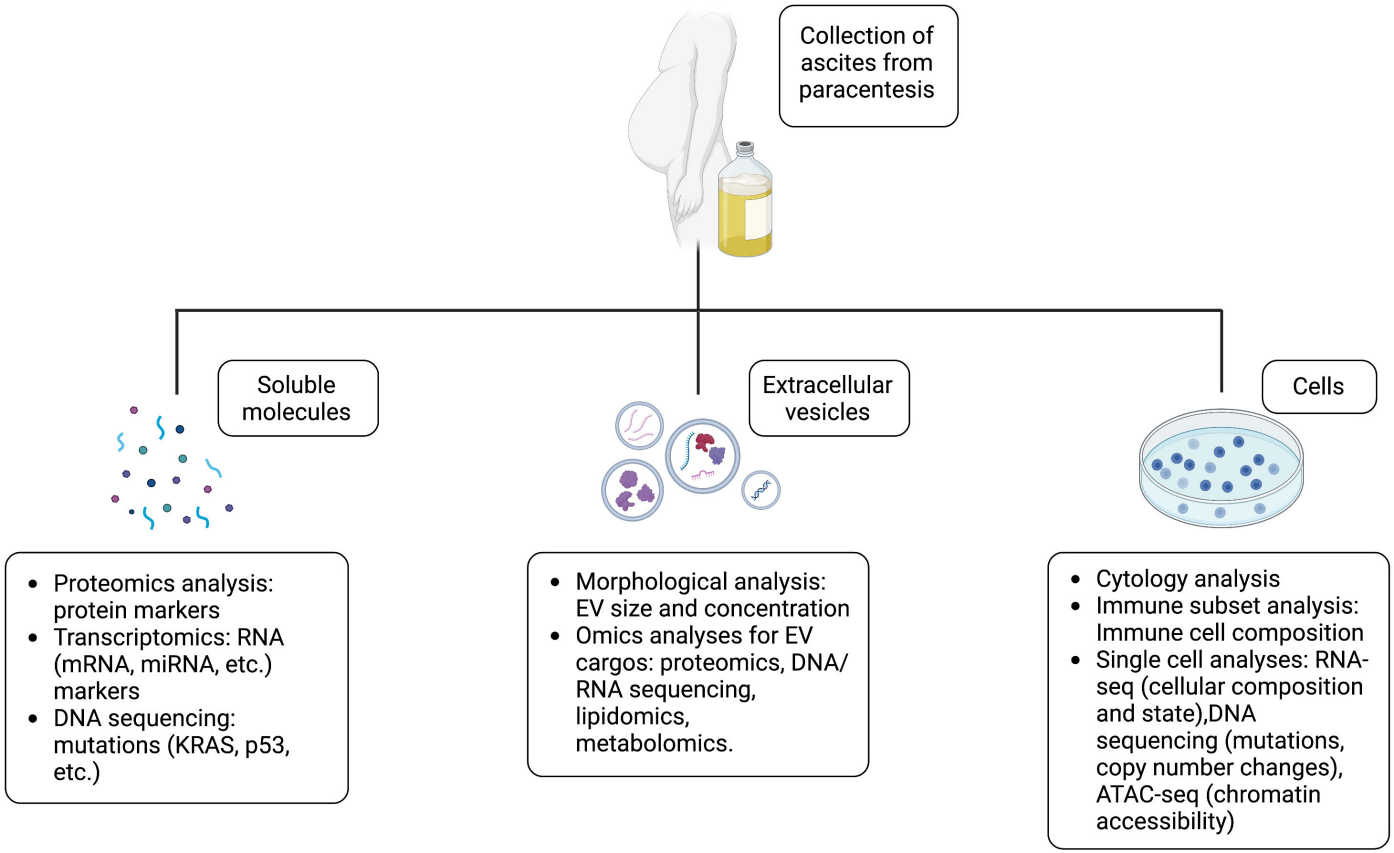
Molecular characterization of malignant ascites in PDAC.

To improve the outcome of PDAC patients with MA, new biomarkers and therapeutic targets are urgently needed for better diagnosis/monitoring of MA and development of more efficacious therapies. Recently, a number of molecular profiling studies aimed at examining the different contents of MA such as soluble molecules (proteins, DNA, and RNA), extracellular vesicles (EVs), and cells have provided new insights into the MA biology and present new opportunities for the development of new biomarkers and therapies (**Figure 1**).

Proteomic analysis of ascites from patients with advanced PDAC or patients with liver cirrhosis by Kitamura et al. revealed 18 malignant ascites-specific proteins. The most frequent were CD13, lymphatic vessel endothelial hyaluronan receptor 1, ficolin-3, and V-set and immunoglobulin domain containing 4 (31). Using high-resolution mass spectrometry Kosanam et al. also identified 816 proteins from MA samples of PDAC patients, 20 of which (membrane or extracellular proteins) were further selected as candidate biomarkers that warrant further validation (32). Detecting gene mutations that are commonly found in PDAC tumors in DNA or cells derived from ascites of PDAC patients has also been explored as a method for diagnosing MA. Using targeted next generation sequencing, Bae et al. found KRAS mutations in cells derived from 5 out of 6 pancreatic cancer MA samples, but none was detected in the 3 ascites samples with suspected malignancy by cytology analysis (tumor cells ≤ 2%) (33). Unfortunately, the KRAS mutational status in the primary tumors for those cases were not reported, hence the accuracy or sensitivity of the detection cannot be established. In an extensive KRAS mutation analysis using PCR amplification of DNA samples isolated from supernatant (cell-free) and cells from ascites fluid as well as primary tumor tissues, Yamashita and colleagues detected KRAS point mutations in 8 out of 9 ascites supernatant samples from patients with pancreatic cancer including 2 cases of negative cytologic diagnosis (34). Direct sequencing also confirmed that the KRAS point mutations detected in the ascites supernatant were identical to those found in ascites cell pellets, microdissected malignant cells from cytologic smears, and primary tumor tissues (34). The proteins and mutations identified in these studies warrant investigation as biomarkers and therapeutic targets in PDAC.

One of the areas that attracted significant attention in the past few years is the characterization of EVs in MA to identify new therapeutic targets and biomarkers. EVs are membrane bound vesicles released into the extracellular space from the cell that contain bioactive molecules such as lipids, proteins, DNA, and RNA. They play important roles in tumor invasion, tumor progression regulation, and neovascularization. Recent studies have shown that EV cargos such as proteins, DNA, and miRNAs can be used as specific markers of PDAC by comparing expression between healthy controls and PDAC patients. For example, higher levels of serum exosomal c-Met and PD-L1 have been correlated with shorter postoperative survival time and thus are possible prognostic factors (35). There have been few studies concerning EVs from ascites fluid of patients with PDAC. Sakaue et al. reported that a higher glycosylation level of CD133 in EVs from ascites could indicate better prognosis for patients with advanced PDAC (36). Proteomic analysis reveals that membrane proteins, glycoproteins, and small GTP binding proteins are enriched in EVs released from PDAC cells and some of them (e.g., CD73) can be detected in EVs derived from MA of PDAC patients (37). Further characterization of EVs in PDAC MA is necessary to explore biomarker and treatment possibilities as done in other cancer types. For example, a phase 1 clinical trial found combining autologous EVs from ascites with granulocyte-macrophage colony stimulating factor to be a possible colorectal cancer treatment (38).

## Current treatment options of malignant ascites in PDAC

Current standard of care for malignant ascites in patients with PDAC focuses solely on the palliation of symptoms (**Table 1**). Common therapies for managing MA include paracenteses and diuretics. While paracentesis is effective in relieving symptoms, it requires repeated treatment which depletes the patients of protein and electrolytes (9). Permanent drains such as the Pluerx and dialysis catheters have been developed to overcome the need of repeated paracentesis. While the permanent drains are an effective alternative to large volume paracentesis, they come with the risk of developing peritonitis, inflammation of the peritoneum due to infection (40). For patients with PDAC, paracentesis provided relief of symptoms in 93% of patients, but only with a mean effect duration of about 10 days (39). Peritoneovenous shunts have also been used to drain MA fluid, but is associated with complications such as shunt occlusion, ascitic leak, and pulmonary edema (57). While not as effective as paracentesis, diuretics such as spironolactone and furosemide have also benefited patients. However, when used in high doses, diuretics can cause systemic blood volume depletion and renal dysfunction (9).

**Table 1:** Treatment options and their outcomes for MA in patients with PDAC.

Treatment Type	Treatment	Treatment Outcome	References
Palliative Therapy	Paracentesis	Short term symptom relief, protein and electrolyte depletion	Smith et al. (39)
	Permanent drains (ex. Pleurx, dialysis catheters)	Technically successful, but many complications	Fleming et al. (40)
	Peritoneovenous shunts	Improvement of abdominal fullness, but postoperative complications significantly decreased overall survival	Tamagawa et al. (41)
Chemotherapy	Hypothermic intraperitoneal chemotherapy (HIPEC)	Resolution of ascites, increased overall survival rate	Valle et al. (42)
	Pressurized intraperitoneal aerosol chemotherapy (PIPAC) combined with nab-paclitaxel	Positive palliative responses, increase in overall survival rate	Ceelen et al. (43), Frassini et al. (44)
	Normothermic intraperitoneal chemotherapy (NIPEC)	Positive palliative responses	Frassini et al. (44)
	Paclitaxel	Decrease in ascites volume, increased control of ascites	Shukuya et al. (45)
	i.p. cisplatin + i.v. gemcitabine + regional hypothermia	Partial response in 13 out of 29 patients, OS of 195 days	Fan et al. (46)
	i.p. paclitaxel + i.v. nab-paclitaxel + i.v. gemcitabine	Increased overall survival, peritoneal recurrence in 75% of patients	Yamada et al. (47)
Targeted Therapy	Catumaxomab	Lengthened puncture-free survival by 35 days when combined with paracentesis	Heiss et al. (48)
	Apatnib/gemcitabine	Increased progression-free survival, no longer required paracentesis	Liang et al. (49)
	i.p. bevacizumab	Well tolerated, improved paracentesis-free survival (albeit, not statistically significant when compared to placebo)	Jordan et al. (50)
	NK4	Targets HGFR, suppressed ascites accumulation in an orthotopic mouse model	Tomoika et al. (51)
Immunotherapy	i.p. nivolumab	No longer required paracentesis, almost no cancer cells in ascites samples	Wang et al. (52)
	Concentrated ascites reinfusion therapy (CART)	Improved symptoms and allowed administration of chemotherapy. Increased levels of IL-12 and IFN-γ	Shirakawa et al. (53) Kobori et al. (54)
	NK cells + PD-1 inhibitor	Activation of multiple immune cell types and elimination of cancer cells	Chen et al. (55)
	i.p. oncolytic therapy	Positive ascites control in 8 of 13 patients	Zhang et al. (56)

i.p., intraperitoneal; i.v., intravenous; OS, overall survival; HGFR, hepatocyte growth factor receptor; NK cells, natural killer cells.

For patients with PC more aggressive therapeutic approaches such as debulking surgery combined with chemotherapy administered at the end of surgery, known as hypothermic intraperitoneal chemotherapy (HIPEC), and pressurized intraperitoneal aerosol chemotherapy (PIPAC) in which diagnostic and staging laparoscopy is combined with an aerosolized drug administered using a high-pressure micro-injection pump have been used in treating MA (**Table 1**). A study employing laparoscopic HIPEC reported complete resolution of ascites in 94% of the 52 patients with PC (42). A Phase I trial of patients with peritoneal metastases of breast, ovarian, and gastrointestinal cancers including pancreatic cancer given PIPAC using nanoparticle albumin bound paclitaxel (NAB-PTX) demonstrated positive responses in 35% of patients with an overall one-year survival rate of 57% (43). Additionally, Frassini and colleagues compared the benefits of cytoreductive surgery combined with HIPEC, PIPAC, or normothermic intraperitoneal chemotherapy (NIPEC) in a meta-analysis of 212 PDAC patients with or without PC. The authors found that patients treated with HIPEC had a favorable 3-year survival rate of 24% compared to 5.3% and 7.9% for those treated with PIPAC and NIPEC, respectively (44).

There have been a handful of reports on small-molecule-based therapies for malignant ascites in patients with PDAC (**Table 1**). Shukuya et al. reported a decrease in ascites volume by 30% and an ascites control rate of 60% when patients were given paclitaxel weekly (Direct intravenous infusion for 1 hour) after failure of gemcitabine (45). Fan et al. reported in a retrospective study that treatment with intraperitoneal cisplatin and intravenous gemcitabine combined with regional hypothermia treatment was well tolerated by pancreatic cancer patients with MA with 13 out of 29 patients demonstrating a partial response and an overall survival of 195 days (46). In a Phase I/II clinical trial, Yamada et al. found that adding intraperitoneal paclitaxel to the standard of care intravenous nab-paclitaxel + gemcitabine led to the disappearance of ascites in 12 out of 30 PDAC patients with PC and a median overall survival of 14.5 months (47). Furthermore, patients who underwent conversion surgery did not reach median survival after 30 months follow-up. Adverse side effects observed include grade 3-4 neutropenia in 70% of the patients (47).

Therapeutic antibodies and proteins have also been explored as treatment options for MA. One example is the trifunctional monoclonal antibody catumaxomab administered by intraperitoneal infusions. Catumaxomab binds to epithelial tumor cells *via* the epithelial cell-adhesion molecule (EpCAM) and T-cells *via* CD3. It also activates Fcγ-receptor I-, IIa- and III-positive accessory cells through its functional Fc domain (58). In a randomized Phase II/III study in patients with epithelial cancers including pancreatic cancer, catumaxomab combined with paracentesis was found to lengthen puncture-free survival by 35 days compared to paracentesis alone (46 vs. 11 days, P < 0.0001) (48). Patients treated with the antibody also had fewer ascites signs and associated symptoms. Inhibiting the VEGF pathway may be beneficial in treating MA. In a case report of a 64-year-old PDAC patient given oral apatinib, which targets VEGF receptor 2, combined with intravenous gemcitabine, Liang et al. described that after 1 month of treatment, the patient no longer needed paracenteses and after 10.5 months of treatment, the patient achieved a progression-free survival for 11 months (49). The antiangiogenic antibody, bevacizumab, which targets the angiogenesis factor VEGF, has also been tested for treating MA in patients with pancreatic cancer. In a Phase II study of intraperitoneal bevacizumab for control of MA in gastrointestinal cancers of which the majority (56%) were patients with pancreatic cancer, Jordan et al. found that the treatment was well tolerated but did not result in statistically significant improvement in paracentesis-free survival compared to the placebo control (14.0 vs. 10.5 days, P = 0.16) (50).

In addition, immunotherapies have been investigated as potential treatment options. Wang et al. recently described a case in which a PDAC patient with MA responded to intraperitoneal nivolumab treatment after failed to respond to intraperitoneal paclitaxel. The amount of MA decreased significantly, and paracenteses was no longer needed after 7 doses (20 mg/dose) of intraperitoneal nivolumab with cancer cells barely detectable in the ascites fluid (52). Other immunotherapies such as cell-free and concentrated ascites reinfusion therapy (CART) have also shown promising results as therapy. Shirakawa et al. reported a positive response to CART in a patient with unresectable pancreatic cancer allowing her to have oral intake of chemotherapy (53). Additionally, cytokine expression profiling indicates that interferon-gamma (IFN-γ) and interleukin 12 levels increased in ascites after CART treatment, which may contribute to the growth inhibition of pancreatic cancer cells (54). This points to their potential as biomarkers for assessing clinical efficacy of CART procedures. Other novel immunotherapy regimens have also been investigated. Chen et al. report a case of combined autologous *ex vivo* expanded natural killer (NK) cells and programmed cell death 1 (PD-1) inhibitor therapy in a pancreatic cancer patient with peritoneal metastasis (55). Promising activity was observed with activation of multiple immune cell types and elimination of cancer cells in the ascites after treatment.

Several other novel approaches have also been explored for treating MA in patients with PDAC. Zhang and colleagues tested an oncolytic virotherapy H101 engineered to specifically target cancer cells with aberrant p53 function in patients with GI cancers (56). Intraperitoneal treatment with H101 was found to induce intraperitoneal immune activation with an increase in both the number of CD8^+^ cells and PD-1 expression in regulatory T cells in the ascites (56). Of the 13 pancreatic cancer patients who received H101, ascites response (>10% reduction in ascites volume) was observed in 5 patients (38.5%) and ascites control (<10% increase in ascites volume) in 8 patients (61.5%). In addition, NK4, an antagonistic peptide against the hepatocyte growth factor receptor, was found to suppress peritoneal dissemination and ascites accumulation in an orthotopic mouse model for PDAC (51). Given that the therapies described above were all only tested in a small number of patients, further studies are needed to validate their clinical efficacy.

## Discussion

MA is an indicator of poor prognosis for patients with PDAC. Positive cytology with low SAAG and increased total protein concentration can be used to diagnose MA, but these methods are not always accurate or sufficiently sensitive. Cell-free ascites DNA analysis has been demonstrated to aid the diagnosis of malignant ascites (34). Current treatments for PDAC patients with ascites focus on palliation with the most common being paracenteses and diuretics. New therapies combining debulking surgery with intraperitoneal chemotherapy such as HIPEC, PIPAC, and NIPEC have shown promising activity in PDAC patients with MA (42, 43).

Biologics including catumaxomab and bevacizumab have demonstrated palliation in gastric and ovarian cancer patients with MA but data on their activity in PDAC patients are sparse. Some successes have also been reported for other therapeutic regimens such as apatinib combined with gemcitabine, intraperitoneal nivolumab, and intraperitoneal oncolytic virotherapy in small studies. Further validation of their clinical utility is needed.

Due to the very limited therapeutic options for treating MA in patients with PDAC, it is imperative to identify new therapeutic targets and biomarkers. Thus, specific analysis of ascites fluid is needed. Some molecular characteristics of ascites recently studied in PDAC including EV cargos are showing some promises. Additional studies focusing on EVs as a potential diagnostic tool and therapeutic target could be very fruitful in bringing about novel approaches for managing MA in patients with PDAC.

In recent years, single cell based genomic tools such as single cell RNA sequencing (scRNA-seq) have been widely used to characterize individual cells in tissues and body fluids such as blood, ascites, and spinal fluids. Several scRNA-seq studies have characterized the cells derived from MA of patients with ovarian, gastric, or colon cancers and revealed a plethora of information on their cellular composition and cell state which could help devise new approaches for the diagnosis and treatment of MA for these cancer types (59–62). For example, scRNA-seq of ascites fluid from gastric cancer revealed dynamic changes of the ascites ecosystem during gastric cancer peritoneal metastasis caused by chemotherapy and immunotherapy providing insight on possible differential treatment strategies (63). Systematic and well-designed single cell-based studies for MA from patients with PDAC are lacking at this point. Similarly, DNA sequencing of MA fluid in PDAC could also reveal differences in mutations between ascites and the primary tumor. Finally, molecular characterization of ascites fluid could also lead to discovery of predictive markers for MA in PDAC and consideration of possible preventive therapies. We believe that such studies could not only significantly help advance our understanding of the biology of MA in PDAC but also lead to new diagnostic markers and therapeutic strategies, potentially making a meaningful impact on the outcome of PDAC patients with MA.

## Author contributions

Both authors designed the review. MH prepared the first draft of the manuscript. All authors contributed to the article and approved the submitted version.
